# Association between complete blood cell count-derived inflammatory indices and left atrial thrombus in patients with nonvalvular atrial fibrillation: a cross-sectional study

**DOI:** 10.3389/fmed.2026.1730240

**Published:** 2026-03-25

**Authors:** You Zhou, Xuewen Song, Jifang Ma, Yuexia Ren, Erpeng Liang, Ke Chen, Weifeng Song, Xiaobiao Zang, Zhihan Zhao, Lei Wang, Yonghui Zhao, Xianqing Wang, Haixia Fu

**Affiliations:** 1 Heart Center of Henan Provincial People’s Hospital, Central China Fuwai Hospital, Central China Fuwai Hospital of Zhengzhou University, Zhengzhou, Henan, China; 2 Department of Hematology, The Affiliated Cancer Hospital of Zhengzhou University & Henan Cancer Hospital, Hemostasis and Thrombosis Diagnostic Engineering Research Center of Henan Province, Zhengzhou, China

**Keywords:** atrial fibrillation, biomarkers, inflammation, risk stratification, thrombosis

## Abstract

**Background:**

Systemic inflammation plays a key role in thrombogenesis, yet the value of routine complete blood count-derived inflammatory indices for predicting left atrial thrombus (LAT) in patients with non-valvular atrial fibrillation (NVAF) remains unclear.

**Methods:**

In this cross-sectional study of 623 patients with NVAF (59 with LAT, 9.5%), we evaluated eight inflammatory indices for their association with LAT, using multivariable logistic regression and receiver operating characteristic (ROC) curve analysis.

**Results:**

Among the inflammatory markers analyzed, neutrophil-to-lymphocyte ratio (NLR), neutrophil-to-mean platelet volume ratio (NMR), systemic immune-inflammation index (SII), systemic inflammation response index (SIRI), and pan-immune-inflammation value (PIV) were significantly associated with LAT risk after multivariable adjustment. ROC curve analysis indicated moderate predictive accuracy for these five indices, with area under the curve values ranging from 0.582 to 0.601, though no significant differences were found between them. Other indices, including monocyte-to-lymphocyte ratio, platelet-to-lymphocyte ratio, and white blood cell-to-mean platelet volume ratio, showed no significant predictive value.

**Conclusion:**

NLR, NMR, SII, SIRI, and PIV are readily accessible and cost-effective inflammatory biomarkers that show association with LAT risk in NVAF patients. These indices may offer supplementary information for risk stratification in clinical practice. Their potential to guide more intensive assessment or therapeutic strategies requires further prospective validation.

## 1 Introduction

Atrial fibrillation (AF), the most prevalent cardiac arrhythmia, affects approximately 60 million individuals globally (1). Systemic thromboembolism, primarily driven by left atrial thrombus (LAT) formation, is a critical complication of nonvalvular AF (NVAF). Despite anticoagulation therapy, LAT is detected in 5.3–8.2% of patients with NVAF undergoing transesophageal echocardiography (TEE) (2–4). The pathogenesis of LAT involves the interplay of blood stasis, endothelial dysfunction, and hypercoagulability (Virchow’s triad), with chronic inflammation serving as a pivotal upstream mediator that promotes both structural remodeling and a prothrombotic state (5–7).

Chronic inflammation promotes thrombogenesis by enhancing pro-coagulant pathways and suppressing physiological anticoagulants (8–11). Blood count-derived inflammatory indices, including neutrophil-to-lymphocyte ratio (NLR), monocyte-to-lymphocyte ratio (MLR), systemic immune-inflammation index (SII), and systemic inflammation response index (SIRI), provide integrated, readily available measures of this inflammatory activity. These and related indices, such as white blood cell-to-mean platelet volume (MPV) ratio (WMR), neutrophil-to-MPV ratio (NMR), platelet-to-lymphocyte ratio (PLR), and pan-immune-inflammation value (PIV), reflect the interplay between inflammatory cells, lymphocytes, and platelets, offering cost-effective insight into the thrombo-inflammatory state.

Recent studies have shown that SII and SIRI are positively correlated with the incidence of paroxysmal AF (12). Furthermore, SII has also emerged as a promising predictor of AF recurrence after ablation (13, 14). Despite the emphasis on comprehensive risk assessment in updated AF management guidelines, current thrombotic risk stratification tools, such as the CHA_2_DS_2_-VASc score, continue to overlook inflammatory dynamics (15). Therefore, despite the recognized role of inflammation in AF, its specific utility in predicting LAT remains underexplored, which constitutes a critical gap in current risk stratification. In this study, eight inflammatory indices were selected to systematically represent distinct yet interconnected pathways of inflammation and thrombosis implicated in AF. This study aimed to assess eight inflammatory indices for LAT prediction, bridging the gap and exploring their potential clinical utility in refining pre-TEE risk assessment and guiding personalized strategies.

## 2 Methods

### 2.1 Study population

**Figure 1:**
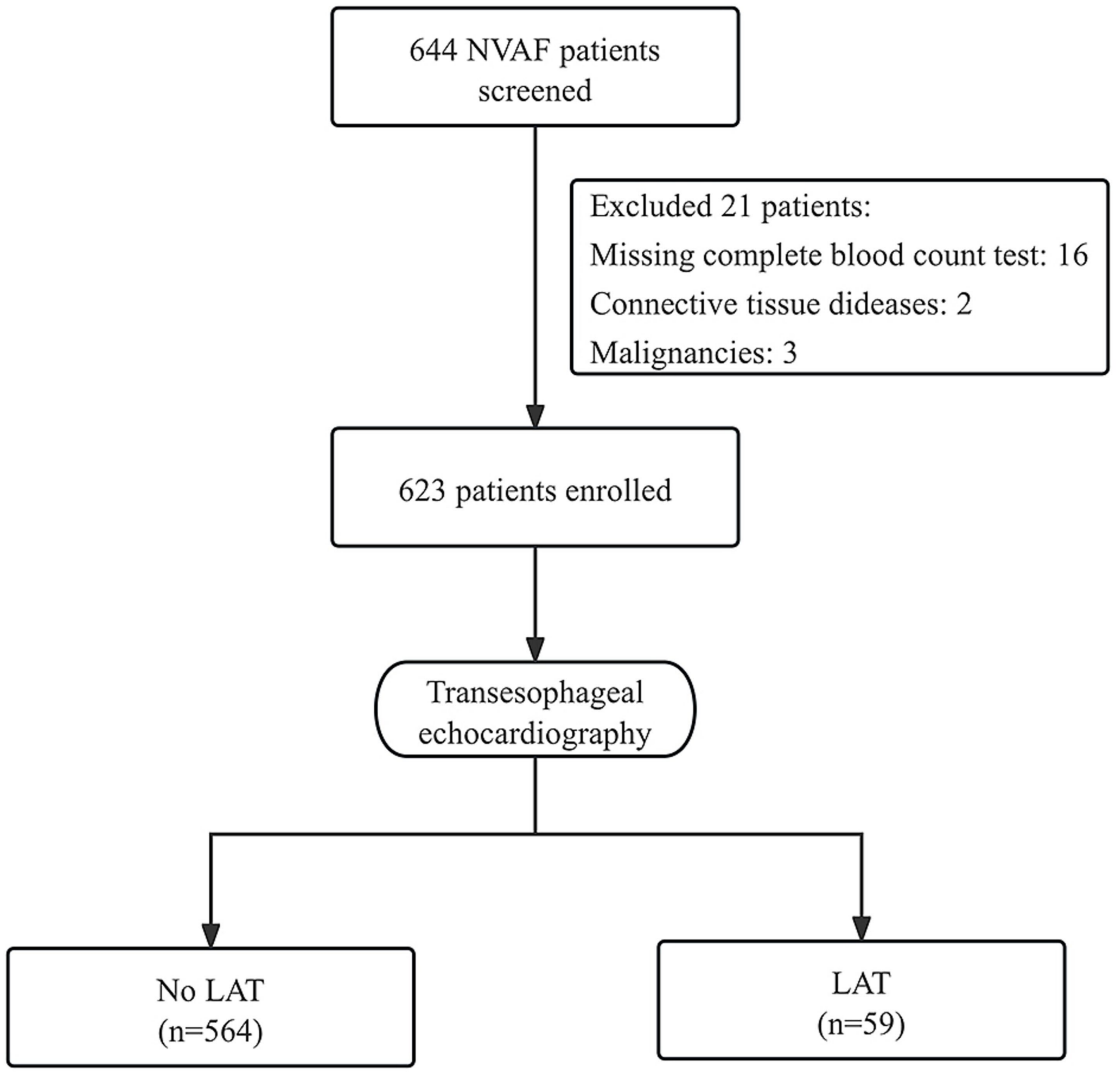
Flow diagram showing screening and recruitment of the study population. NVAF, nonvalvular atrial fibrillation; LAT, left atrial thrombus.

This study retrospectively included 644 patients with NVAF who were scheduled for catheter ablation and underwent pre-procedural TEE at Fuwai Central China Cardiovascular Hospital and Henan Provincial People’s Hospital from January 2015 to April 2025. The methodology aligns with that of a prior publication (16). NVAF was defined as AF in the absence of moderate or severe mitral stenosis or mechanical heart valve prostheses (15). First-diagnosed AF included those with no previous AF diagnosis, regardless of symptom duration or severity (15). Exclusion criteria included a history of malignancy, connective tissue disorders, and incomplete blood count data. After exclusions, 623 participants were included (Figure 1). The study received approval from the Ethics Committees of both institutions (Approval No.: 24–98; Date: 2024-12-06). Due to the retrospective design, informed consent was waived. This study was conducted in accordance with the Strengthening the Reporting of Observational Studies in Epidemiology (STROBE) guidelines. A completed STROBE checklist is provided as Supplementary Table S1.

### 2.2 Data collection

Demographic and clinical data—including age, sex, comorbidities (coronary artery disease, hypertension, heart failure, AF type, diabetes, stroke), echocardiographic measures, laboratory results, and medication use (oral anticoagulants, beta-blockers, diuretics, renin–angiotensin system inhibitors)—were collected from medical records prior to TEE.

### 2.3 Biochemical analyses

Fasting venous blood samples were obtained from all patients on the day preceding TEE. Analyses were conducted in the core laboratories of the participating hospitals using standardized procedures. Glomerular filtration rate was estimated via the Cockcroft–Gault formula. Body mass index was computed as weight divided by height squared (kg/m^2^).

### 2.4 Inflammation indices calculation

Inflammatory markers were derived from the initial blood test after admission. The indices were calculated as follows:

NLR: neutrophil count ÷ lymphocyte count.
MLR: monocyte count ÷ lymphocyte count.
WMR: white blood cell count ÷ MPV.
NMR: neutrophil count ÷ MPV.
PLR: platelet count ÷ lymphocyte count.
SII: (neutrophil count × platelet count) ÷ lymphocyte count.
SIRI: (neutrophil count × monocyte count) ÷ lymphocyte count.
PIV: (neutrophil count × platelet count × monocyte count) ÷ lymphocyte count.

### 2.5 Echocardiographic data

TEE was conducted by two certified physicians to screen for thrombus formation in the left atrium. LAT was identified as a clearly demarcated, echogenic mass distinct from the atrial wall in texture and density (17). Left atrial diameter and left ventricular ejection fraction were assessed via M-mode or two-dimensional imaging in the parasternal long-axis view. Disagreements were resolved through consensus or third-party expert review.

### 2.6 *Post-hoc* power analysis

Given the retrospective design, a *post-hoc* power analysis was performed to assess the statistical sufficiency of the sample size for the primary findings. Using the observed effect size [odds ratio (OR) = 2.113] for the NLR—the index with the highest discriminatory area under the curve (AUC) in our study—along with the actual case (*n* = 59) and control (*n* = 564) numbers, and a two-sided alpha of 0.05, the analysis indicated a statistical power of approximately 85%. This exceeds the conventional 80% threshold, indicating that the study was adequately powered to detect the observed association for the primary biomarker.

### 2.7 Statistical analysis

Continuous data are expressed as mean ± standard deviation. Student’s *t*-tests and the Mann–Whitney U test were applied to compare variables with normal and non-normal distributions, respectively. Categorical variables are presented as percentages and compared using chi-square tests. Restricted cubic splines with knots at the 5th, 35th, 65th, and 95th percentiles evaluated dose–response relationships between inflammatory indices and LAT risk. Covariates for the multivariable logistic regression models were selected *a priori* based on established clinical and pathophysiological relevance to thrombotic risk in AF (5, 15). Univariable analysis was performed initially, followed by sequential multivariable adjustments: Model 1 adjusted for basic demographic factors (age and sex); Model 2 additionally included established clinical risk factors for thrombosis (coronary heart disease, heart failure, hypertension, diabetes, and stroke); Model 3 further adjusted for AF-specific and treatment-related variables (type of AF, oral anticoagulant use, left atrial diameter and left ventricular ejection fraction). This stepped approach allowed for assessment of the independent association of inflammatory indices with LAT beyond conventional risk determinants. A complete-case analysis was used; participants with missing data for any model variable were excluded from that analysis. Results are reported as OR with 95% confidence intervals (CI). Receiver operating characteristic (ROC) curves established optimal cutoff values of inflammatory indices for predicting LAT; AUC values were compared pairwise. A *p*-value < 0.05 indicated statistical significance. Analyses were conducted using SPSS Statistics 27.0 and R 4.4.2.

## 3 Results

### 3.1 Patient characteristics

**Figure 2:**
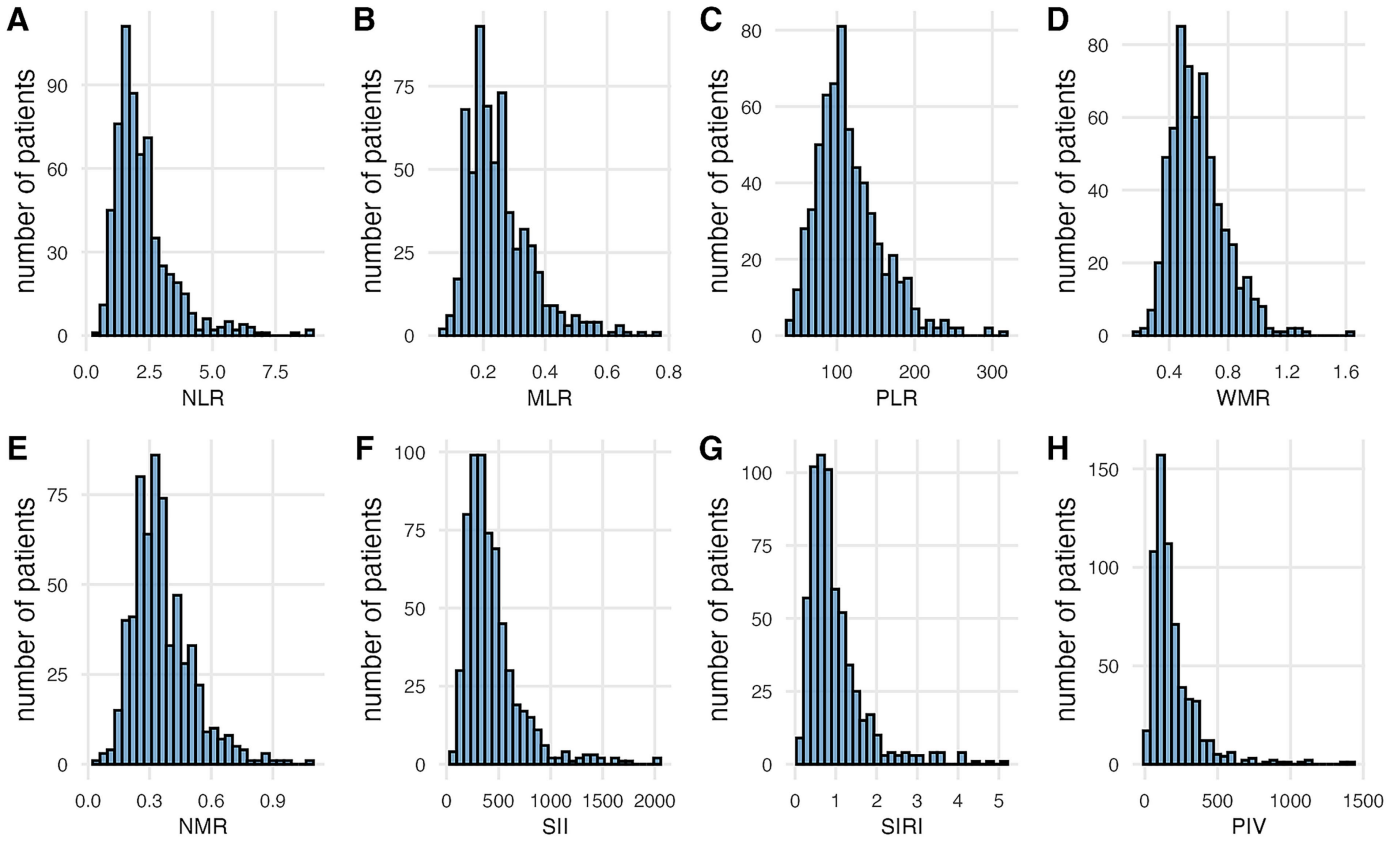
Distributions of the inflammatory indices among the enrolled patients **(A–H)**. NLR, Neutrophil-to-lymphocyte ratio; MLR, monocyte-to-lymphocyte ratio; PLR, platelet-to-lymphocyte ratio; WMR, white blood cell-to-mean platelet volume ratio; NMR, neutrophil-to-mean platelet volume ratio; SII, systemic immune-inflammation index; SIRI, systemic inflammation response index; PIV, pan-immune-inflammation value.

This study enrolled 623 patients with NVAF, of whom 59 (9.5%) had LAT. The cohort had a mean age of 62.17 ± 9.92 years, with 67.45% male participants. Among the participants, 33.5% received oral anticoagulants at the time of admission. Compared with non-LAT patients, those with LAT exhibited significantly higher rates of persistent AF, lower left ventricular ejection fraction, and elevated white blood cell and neutrophil counts. Among the eight analyzed inflammatory biomarkers, NLR, NMR, SII, SIRI, and PIV were significantly elevated in the LAT group, whereas MLR, PLR, and WMR showed no intergroup differences (Table 1). The distribution patterns of inflammatory indices are shown in Figure 2.

**Table 1:** Baseline characteristics of the study population stratified by left atrial thrombus.

Characteristics	Total (*n* = 623)	No left atrial thrombus (*n* = 564)	left atrial thrombus (*n* = 59)	*p*-value
Demographics and clinical characteristics				
Male, *n* (%)	420 (67.4%)	380 (67.4%)	40 (67.8%)	0.948
Age (years)	62.17 ± 9.92	62.17 ± 9.92	59.76 ± 11.1	0.113
Body mass index (Kg/m^2^)	26.23 ± 3.58	26.18 ± 3.58	26.77 ± 3.58	0.232
Paroxysmal atrial fibrillation, *n* (%)	226 (36.3%)	212 (37.6%)	14 (23.7%)	0.035
First-diagnosed atrial fibrillation, *n* (%)	366 (58.7%)	330 (58.5%)	36 (61.0%)	0.291
Comorbidities and medications				
Coronary heart diseases, *n* (%)	137 (22.0%)	129 (22.9%)	8 (13.6%)	0.100
Heart failure, *n* (%)	156 (25.0%)	142 (25.2%)	14 (23.7%)	0.807
Hypertension, *n* (%)	297 (47.7%)	274 (48.6%)	23 (39.0%)	0.160
Diabetes, *n* (%)	137 (22.0%)	129 (22.9%)	8 (13.6%)	0.100
Stroke, *n* (%)	103 (16.5%)	94 (16.7%)	9 (15.3%)	0.781
Oral anticoagulant, *n* (%)	209 (33.5%)	190 (33.7%)	19 (32.2%)	0.818
Beta-blockers, *n* (%)	232 (37.2%)	213 (37.8%)	19 (32.2%)	0.400
Renin-angiotensin system inhibitors, *n* (%)	140 (22.5%)	127 (22.5%)	13 (22.0%)	0.932
Spironolactone, *n* (%)	88 (14.1%)	81 (14.4%)	7 (11.9%)	0.600
Echocardiographic and laboratory parameters				
Left atrium diameter, mm	44.91 ± 7.74	44.86 ± 7.81	45.45 ± 7.06	0.557
Left ventricular ejection fraction, %	55.90 ± 9.91	56.35 ± 9.61	51.63 ± 12.44	0.008
Estimated glomerular filtration rate, mL/min/1.73 m^2^	85.77 ± 20.10	85.37 ± 20.25	89.52 ± 18.28	0.132
White blood cell count, (×10^9^/L)	6.06 ± 1.67	6.02 ± 1.69	6.38 ± 1.48	0.046
Neutrophil count, (×10^9^/L)	3.67 ± 1.38	3.63 ± 1.39	4.02 ± 1.28	0.007
Lymphocytes count, (×10^9^/L)	1.80 ± 0.58	1.81 ± 0.59	1.77 ± 0.49	0.900
Monocyte count, (×10^9^/L)	0.42 ± 0.14	0.42 ± 0.14	0.43 ± 0.12	0.319
Hemoglobin, (g/L)	138.26 ± 17.27	137.88 ± 17.35	141.86 ± 16.08	0.076
Platelet count, (×10^9^/L)	194.77 ± 57.52	194.34 ± 57.26	198.81 ± 60.33	0.528
Calculated inflammatory indices				
Neutrophil-to-lymphocyte ratio	2.23 ± 1.17	2.21 ± 1.19	2.42 ± 0.95	0.010
Monocyte-to-lymphocyte ratio	0.25 ± 0.10	0.25 ± 0.10	0.26 ± 0.10	0.485
Platelet-to-lymphocyte ratio	115.71 ± 41.87	115.62 ± 42.59	116.63 ± 34.52	0.355
White blood cell-to-mean platelet volume ratio	0.60 ± 0.19	0.59 ± 0.19	0.63 ± 0.19	0.126
Neutrophil-to-mean platelet volume ratio	0.36 ± 0.15	0.36 ± 0.14	0.40 ± 0.15	0.024
Systemic immune-inflammation index	434.13 ± 270.03	429.50 ± 272.39	478.41 ± 244.01	0.014
Systemic inflammation response index	0.97 ± 0.70	0.96 ± 0.70	1.07 ± 0.63	0.032
Pan-immune-inflammation value	192.96 ± 167.68	190.82 ± 169.90	213.41 ± 144.44	0.038

### 3.2 Dose–response relationship between inflammatory indices and risk for LAT

**Figure 3:**
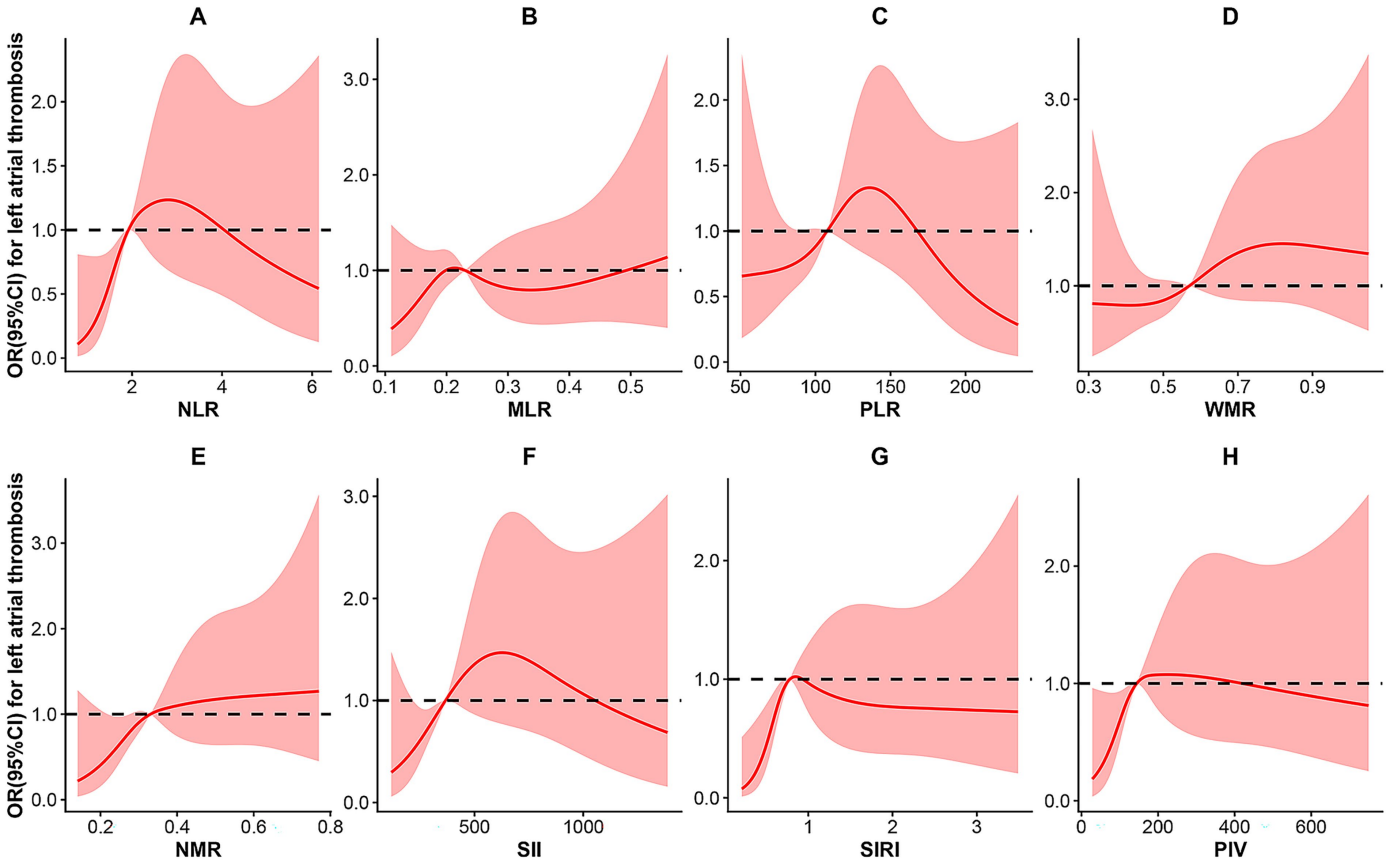
Dose–response curve of inflammatory indices and the risk for left atrial thrombus. **(A)** NLR; **(B)** MLR; **(C)** PLR; **(D)** WMR; **(E)** NMR; **(F)** SII; **(G)** SIRI; **(H)** PIV. NLR, neutrophil-to-lymphocyte ratio; MLR, monocyte-to-lymphocyte ratio; PLR, platelet-to-lymphocyte ratio; WMR, white blood cell-to-mean platelet volume ratio; NMR, neutrophil-to-mean platelet volume ratio; SII, systemic immune-inflammation index; SIRI, systemic inflammation response index; PIV, pan-immune-inflammation value; OR, odds ratio; CI, confidence interval.

To further explore the dose–response relationship between inflammatory indices and the risk of LAT, restricted cubic spline models with predefined knots were applied (Figure 3). A significant non-linear association was observed between NLR and LAT risk (P for non-linearity = 0.032). The spline curve demonstrated a steep increase in the odds of LAT at lower NLR values, followed by a gradual attenuation and subsequent decline at higher levels, suggesting a threshold effect rather than a simple linear relationship. In contrast, for the remaining seven indices, *p*-values for non-linearity all exceeded 0.05, indicating that a linear model provided an adequate fit to the data for these markers.

### 3.3 Logistic regression analysis of the association between inflammatory indices and LAT

Regression analyses revealed significant associations between specific inflammatory indices and the LAT risk in patients with NVAF (Table 2 and Supplementary Table S2). When analyzed as continuous variables, NLR, NMR, SIRI, and PIV consistently predicted an elevated risk for LAT across the adjusted models. NLR exhibited robust associations, with ORs increasing from 1.897 (*p* = 0.028) in the unadjusted model to 2.594 (*p* = 0.003) after adjusting for clinical thrombosis factors (Model 2), and remained significant in the fully adjusted models [OR = 2.113 (95% CI: 1.087–4.108), *p* = 0.027]. NMR demonstrated a strong association, with ORs increasing to 2.800 (*p* = 0.005) in Model 2 and sustained significance in Model 3 [OR = 2.288 (95% CI: 1.061–4.933), *p* = 0.035]. The SII and SIRI showed progressive risk elevation, although the association of the SII was attenuated in Model 3 [OR = 1.635 (95% CI: 0.942–2.838), *p* = 0.081], while the SIRI remained robust [OR = 1.858 (95% CI: 1.133–3.049), *p* = 0.014]. PIV showed modest but consistent associations [OR = 1.533 (95% CI: 1.010–2.326), *p* = 0.045; Model 3].

**Table 2:** Association between inflammatory indices (as continuous variables) and left atrial thrombus.

Inflammatory indices	Unadjusted model		Model 1		Model 2		Model 3	
	OR (95% CI)	*p*-value	OR (95% CI)	*p*-value	OR (95% CI)	*p*-value	OR (95% CI)	*p*-value
NLR	1.897 (1.070–3.363)	0.028	2.250 (1.236–4.098)	0.008	2.594 (1.393–4.832)	0.003	2.113 (1.087–4.108)	0.027
MLR	1.406 (0.702–2.812)	0.336	1.685 (0.807–3.517)	0.165	1.895 (0.887–4.049)	0.099	1.995 (0.881–4.522)	0.098
PLR	1.246 (0.580–2.678)	0.573	1.351 (0.617–2.956)	0.451	1.415 (0.641–3.127)	0.390	1.070 (0.463–2.475)	0.874
WMR	2.010 (0.825–4.897)	0.125	2.053 (0.836–5.042)	0.117	2.343 (0.944–5.814)	0.066	1.972 (0.747–5.201)	0.170
NMR	2.276 (1.131–4.580)	0.021	2.427 (1.195–4.930)	0.014	2.800 (1.357–5.774)	0.005	2.288 (1.061–4.933)	0.035
SII	1.695 (1.037–2.770)	0.035	1.815 (1.099–2.996)	0.020	2.001 (1.197–3.344)	0.008	1.635 (0.942–2.838)	0.081
SIRI	1.598 (1.037–2.464)	0.034	1.774 (1.133–2.778)	0.012	1.985 (1.248–3.157)	0.004	1.858 (1.133–3.049)	0.014
PIV	1.479 (1.015–2.154)	0.041	1.544 (1.054–2.261)	0.026	1.672 (1.131–2.472)	0.010	1.533 (1.010–2.326)	0.045

Model 1: Adjusted for age and gender.

Model 2: Additionally adjusted for clinical thrombosis factors (coronary heart disease, heart failure, hypertension, diabetes, stroke).

Model 3: Further adjusted for paroxysmal atrial fibrillation, oral anticoagulant use, left atrial diameter, and left ventricular ejection fraction.

All inflammation indices were natural logarithm (ln) transformed.

OR, odds ratio; CI, confidence interval; NLR, neutrophil-to-lymphocyte ratio; MLR, monocyte-to-lymphocyte ratio; PLR, platelet-to-lymphocyte ratio; WMR, white blood cell-to-mean platelet volume ratio; NMR, neutrophil-to-mean platelet volume ratio; SII, systemic immune-inflammation index; SIRI, systemic inflammation response index; PIV, pan-immune-inflammation value.

Tertile-based analyses confirmed dose–response relationships for NLR, NMR, and SII, with the highest tertiles showing significantly elevated risks and significant trend tests (P for trend <0.05). The SIRI demonstrated significantly elevated risks in the highest tertile across all models despite non-significant trend tests (P for trend = 0.054). PIV showed borderline significance in the highest tertile [OR = 2.070 (95% CI: 0.961–4.460) in Model 3, *p* = 0.063], with a near-significant trend (P for trend = 0.069). In contrast, the MLR, PLR, and WMR showed no significant associations in any of the models (*p* > 0.05).

### 3.4 Discriminatory ability of inflammatory indices

**Figure 4:**
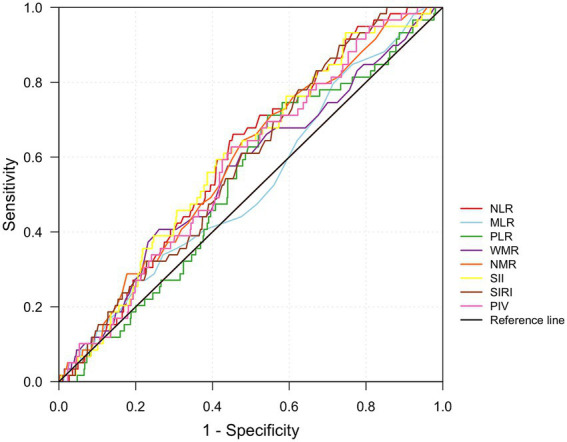
Receiver operating characteristic curves for inflammatory indices as predictors of left atrial thrombus. NLR, neutrophil-to-lymphocyte ratio; MLR, monocyte-to-lymphocyte ratio; PLR, platelet-to-lymphocyte ratio; WMR, white blood cell-to-mean platelet volume ratio; NMR, neutrophil-to-mean platelet volume ratio; SII, systemic immune-inflammation index; SIRI, systemic inflammation response index; PIV, pan-immune-inflammation value.

ROC curve analysis was used to evaluate the predictive performance of the eight inflammatory indices for LAT risk (Figure 4). NLR, NMR, SII, SIRI, and PIV demonstrated statistically significant differences (*p* < 0.05). NLR showed the highest AUC [0.601 (95% CI: 0.534–0.668), *p* = 0.01], followed by SII [AUC = 0.598 (95% CI: 0.528–0.667), *p* = 0.014], NMR [AUC = 0.589 (95% CI: 0.518–0.661), *p* = 0.024], SIRI [AUC = 0.585 (95% CI: 0.517–0.652), *p* = 0.032] and PIV [AUC = 0.582 (95% CI: 0.512–0.652), *p* = 0.038] (Table 3). The MLR, PLR, and WMR lacked predictive significance (*p* > 0.05). Pairwise comparisons of AUCs revealed no statistically significant differences among NLR, NMR, SII, SIRI, and PIV (all *p* > 0.05), indicating comparable discriminatory abilities (Table 4).

**Table 3:** Discriminatory ability of inflammatory indices.

Inflammatory indices	Cutoff value	Sensitivity (%)	Specificity (%)	AUC (95% CI)	*p*-value
NLR	2.00	66.1	54.6	0.601 (0.534–0.668)	0.010
MLR	0.19	79.7	31.4	0.528 (0.452–0.603)	0.485
PLR	104.44	71.2	45.7	0.537 (0.464–0.609)	0.355
WMR	0.68	59.3	75	0.560 (0.482–0.639)	0.126
NMR	0.31	72.9	44	0.589 (0.518–0.661)	0.024
SII	253.60	93.2	25.4	0.598 (0.528–0.667)	0.014
SIRI	0.57	86.4	30.7	0.585 (0.517–0.652)	0.032
PIV	150.91	62.7	55.1	0.582 (0.512–0.652)	0.038

AUC, area under the curve; CI, confidence interval; NLR, neutrophil-to-lymphocyte ratio; MLR, monocyte-to-lymphocyte ratio; PLR, platelet-to-lymphocyte ratio; WMR, white blood cell-to-mean platelet volume ratio; NMR, neutrophil-to-mean platelet volume ratio; SII, systemic immune-inflammation index; SIRI, systemic inflammation response index; PIV, pan-immune-inflammation value.

**Table 4:** Pairwise comparison of the AUCs among inflammatory indices.

Comparison	AUC	
	Difference (95% CI)	*p*-value
NLR—NMR	0.012 (−0.063, 0.086)	0.756
NLR—SII	0.004 (−0.048, 0.056)	0.889
NLR—SIRI	0.016 (−0.024, 0.057)	0.427
NLR—PIV	0.019 (−0.040, 0.079)	0.525
NMR—SII	−0.008 (−0.059, 0.042)	0.752
NMR—SIRI	0.005 (−0.063, 0.072)	0.895
NMR—PIV	0.008 (−0.040, 0.055)	0.757
SII—SIRI	0.013 (−0.043, 0.069)	0.657
SII—PIV	0.016 (−0.017, 0.048)	0.341
SIRI—PIV	0.003 (−0.037, 0.043)	0.883

CI, confidence interval; NLR, neutrophil-to-lymphocyte ratio; NMR, neutrophil-to-mean platelet volume ratio; SII, systemic immune-inflammation index; SIRI, systemic inflammation response index; PIV, pan-immune inflammation value.

## 4 Discussion

This study demonstrated that specific inflammatory indices (NLR, NMR, SII, SIRI, and PIV) are independently associated with the presence of LAT in patients with NVAF, whereas the MLR, PLR, and WMR lacked discriminatory ability. Given the cross-sectional design, we cannot exclude the possibility of reverse causation, wherein thrombus presence may itself amplify systemic inflammation. The discriminative accuracy of the five significant indices was moderate (AUC range: 0.58–0.60) in this retrospective analysis. While these AUC values indicate limited standalone diagnostic performance, the routine availability and cost-effectiveness of these indices support their potential role as adjunct screening tools for thrombotic risk stratification, particularly in guiding decisions for further imaging assessment.

The association between an elevated NLR and thrombotic risk is mechanistically rooted in neutrophilic inflammation, which can drive endothelial dysfunction, platelet activation, and a prothrombotic state (8). Our findings confirm the established link between an elevated NLR and thrombotic risk in AF, aligning with prior evidence (18, 19). Importantly, this study extends current knowledge by identifying the NMR, SII, SIRI, and PIV as significant predictors. Unlike NLR, these composite indices capture broader pathophysiology. NMR integrates neutrophilic inflammation with platelet activation via MPV (20), while SII and SIRI reflect synergistic interactions between inflammation, platelets, and monocytes. Although NLR demonstrated the highest predictive AUC, SII and SIRI exhibited comparable discriminative power and greater robustness in multivariable models, likely owing to their composite nature. In contrast, PIV showed lower effect sizes, potentially diluted by the inclusion of diverse inflammatory parameters. The moderate AUC values (0.58–0.60) indicate that, while statistically significant, the standalone diagnostic accuracy of these indices remains limited. However, their utility as sensitive screening tools to identify high-risk patients warranting advanced imaging is noteworthy.

In contrast to previous reports on MLR and PLR in oncology (21, 22), our study found no significant association between these indices and LAT risk. This discrepancy likely reflects distinct disease pathophysiology. MLR and PLR are markers of monocyte and platelet activity within the tumor microenvironment (23), whereas LAT formation in AF is primarily driven by hemodynamic stasis and neutrophil-mediated endothelial injury. The WMR has limited biological specificity, as its inclusion of total white blood cell count dilutes the specificity for isolating neutrophil-driven inflammation or platelet activation – key pathways in thrombogenesis. Furthermore, its broad composition may fail to capture the synergistic interplay between specific immune subsets reflected by NLR or SIRI. The lack of statistical significance for WMR in our cohort underscores the need for biomarkers with clearer mechanistic links to atrial endothelial dysfunction and hypercoagulability.

Our findings resonate with extensive literature on the prognostic value of inflammatory indices in cardiovascular diseases while underscoring the distinct pathophysiology of LAT formation in AF. An umbrella review reported a significant association between elevated SII and major adverse cardiovascular events in coronary artery disease (OR = 2.36), paralleling our observation for LAT (24). Comparisons with left ventricular thrombus (LVT) research are also informative. In patients with acute myocardial infarction, inflammatory markers are associated with LVT presence (25), and a higher NLR is linked to less LVT resolution despite anticoagulation (26). A large study found no significant difference in embolic risk between ischemic and non-ischemic cardiomyopathies once a thrombus formed (27), aligning with the concept that inflammation is a common risk factor across etiologies. However, the location and hemodynamic environment differ substantially between left atrial appendage and ventricular thrombi. Consequently, the predictive efficacy and optimal cut-off values of these inflammatory indices likely vary by thrombus type, a possibility that warrants dedicated investigation.

These findings extend beyond simple statistical associations to offer clinical support for the central role of “thromboinflammation” in AF-related thrombogenesis (28). Elevated NLR and SII are not merely markers of systemic inflammation but also surrogate indicators of interconnected cellular pathways that drive clot formation. Specifically, a high NLR signifies neutrophilia and relative lymphopenia, which may reflect excessive formation of neutrophil extracellular traps (NETs). NETs provide a scaffold for thrombi, expose procoagulant histones, and potently activate platelets and the coagulation cascade, directly linking innate immune response to hypercoagulability (29). Moreover, the significant association of SII and PIV, which incorporate platelet counts, likely captures enhanced platelet-leukocyte interaction. Activated platelets bind to monocytes and neutrophils via surface molecules like P-selectin, forming aggregates. This interaction stimulates monocytes to express tissue factor, thereby initiating the extrinsic coagulation pathway (30). These routine blood count-derived indices capture the continuum from inflammatory cell activation and dysregulation to platelet hyperactivity and a prothrombotic state. Thus, they can stratify LAT risk even in the presence of concomitant atrial stasis.

To illustrate a potential clinical application, these inflammatory indices could be integrated into a stepped assessment protocol. For instance, in an NVAF patient with an intermediate CHA_2_DS_2_-VASc score, an elevated NLR or SII might prompt a more definitive investigation, such as TEE, to rule out LAT. Furthermore, while the CHA_2_DS_2_-VASc score captures clinical thromboembolic risk factors, it does not incorporate inflammatory activity. The indices identified in our study reflect distinct pathophysiological pathways and thus may provide complementary information. Future studies should formally evaluate whether combining these readily available inflammatory markers with clinical risk scores improves the predictive accuracy for LAT and optimizes patient-specific management strategies.

Importantly, this inflammation-mediated pathway is not merely a bystander but a modifiable therapeutic target, which aligns with the clinical relevance of our findings. The observation that NLR can be reduced by interleukin-targeting therapies (e.g., canakinumab, ziltivekimab) and that colchicine can reduce recurrent ischemic stroke in AF patients, provides a compelling translational link (31–34). Our findings suggest a dual role for the identified inflammatory indices (NLR, SII, SIRI, PIV). First, they are accessible tools for identifying NVAF patients with heightened thromboinflammation who may benefit from intensified monitoring or therapy. Second, they serve as potential biomarkers for monitoring the efficacy of anti-inflammatory interventions aimed at reducing thrombotic risk. Our study mechanistically links specific cellular interactions, such as platelet-leukocyte cross-talk, to clinical thrombotic outcomes. Therefore, it strengthens the rationale for incorporating these low-cost indices into integrated risk models and for exploring inflammation-modulating strategies in AF management.

This study has several limitations. First, its retrospective, cross-sectional design precludes causal inference and provides only single-time-point measurements for inflammatory indices. Second, the moderate discriminative ability of the indices, along with a *post-hoc* power analysis that likely overestimates adequacy due to its reliance on the maximum observed OR, suggests that our study is exploratory and may be underpowered to detect more modest associations. Third, our cohort consisted specifically of NVAF patients scheduled for ablation, which may limit generalizability to other clinical populations. Fourth, despite adjustment for a range of clinical covariates, residual confounding cannot be excluded as data on specific inflammatory mediators such as interleukin-6 and coagulation biomarkers such as D-dimer were unavailable. Fifth, the lack of detailed data on anticoagulant type, dosage, duration, and adherence further limits the clinical interpretability of our findings regarding thrombus presence under therapy. Additionally, the robustness of our findings could be further strengthened by sensitivity analyses, such as excluding patients on anticoagulation or using alternative model specifications. The absence of such analyses highlights an important avenue for future validation. Finally, while TEE has high diagnostic accuracy, microthrombi (<2 mm), particularly in the complex left atrial appendage, could be missed (17).

## 5 Conclusion

This study found that the NLR, NMR, SII, SIRI, and PIV were associated with LAT in patients with NVAF. However, given their moderate predictive accuracy, their clinical utility as standalone predictors is limited. These preliminary findings warrant future prospective studies to validate their association and to investigate whether integrating these indices with established clinical scores can improve risk assessment in patients with NVAF.

## Data Availability

The raw data supporting the conclusions of this article will be made available by the authors, without undue reservation.
