# Supplementary material for: Association between complete blood cell count-derived inflammatory indices and left atrial thrombus in patients with nonvalvular atrial fibrillation: a cross-sectional study
Source: Front Med (Lausanne). 2026 Mar 25;13:1730240. doi: 10.3389/fmed.2026.1730240 (PMC13059205; doi:10.3389/fmed.2026.1730240)
Supplement: Supplementary file 1 [file Supplementary_file_1.docx]

Supplementary Table S1. Tertile-based analysis of the association between inflammatory indices and left atrial thrombus.

| Indices | Tertile | Unadjusted Model  OR (95% CI) | Model 1  OR (95% CI) | Model 2  OR (95% CI) | Model 3  OR (95% CI) | P for trend  (Model 3) |
| --- | --- | --- | --- | --- | --- | --- |
| **NLR** | 1(Ref) | 1.00 | 1.00 | 1.00 | 1.00 | **0.033** |
|  | 2 | 1.400 (0.682–2.874) | 1.514 (0.732–3.129) | 1.577 (0.760–3.272) | 1.794 (0.830–3.880) |  |
|  | 3 | **1.980 (1.002–3.909)** | **2.427 (1.188–4.957)** | **2.681 (1.302–5.522)** | **2.345 (1.075–5.116)** |  |
| **MLR** | 1(Ref) | 1.00 | 1.00 | 1.00 | 1.00 | 0.180 |
|  | 2 | 1.061 (0.540–2.085) | 1.159 (0.584–2.299) | 1.182 (0.591–2.364) | 0.928 (0.436–1.974) |  |
|  | 3 | 1.255 (0.652–2.417) | 1.471 (0.736–2.942) | 1.602 (0.788–3.255) | 1.654 (0.778–3.516) |  |
| **PLR** | 1(Ref) | 1.00 | 1.00 | 1.00 | 1.00 | 0.658 |
|  | 2 | **1.969 (0.997–3.889)** | **2.011 (1.013–3.992)** | 1.963 (0.986–3.909) | 1.698 (0.832–3.463) |  |
|  | 3 | 1.386 (0.675–2.844) | 1.489 (0.718–3.091) | 1.507 (0.724–3.134) | 1.210 (0.562–2.601) |  |
| **WMR** | 1(Ref) | 1.00 | 1.00 | 1.00 | 1.00 | 0.225 |
|  | 2 | 0.913 (0.452–1.843) | 0.901 (0.445–1.825) | 0.938 (0.461–1.909) | 0.964 (0.454–2.045) |  |
|  | 3 | 1.465 (0.773–2.774) | 1.449 (0.763–2.753) | 1.548 (0.823–3.036) | 1.519 (0.751–3.072) |  |
| **NMR** | 1(Ref) | 1.00 | 1.00 | 1.00 | 1.00 | **0.045** |
|  | 2 | 2.042 (0.987–4.221) | **2.095 (1.009–4.347)** | **2.269 (1.088–4.735)** | 1.985 (0.906–4.351) |  |
|  | 3 | **2.130 (1.035–4.384)** | **2.244 (1.085–4.643)** | **2.534 (1.213–5.292)** | **2.269 (1.038–4.962)** |  |
| **SII** | 1(Ref) | 1.00 | 1.00 | 1.00 | 1.00 | **0.038** |
|  | 2 | 1.492 (0.717–3.107) | 1.540 (0.737–3.219) | 1.611 (0.768–3.379) | 1.469 (0.679–3.176) |  |
|  | 3 | **2.238 (1.120–4.472)** | **2.388 (1.187–4.802)** | **2.686 (1.322–5.455)** | **2.175 (1.036–4.567)** |  |
| **SIRI** | 1(Ref) | 1.00 | 1.00 | 1.00 | 1.00 | 0.054 |
|  | 2 | **2.309 (1.132–4.711)** | **2.518 (1.220–5.195)** | **2.630 (1.269–5.450)** | **2.380 (1.100–5.148)** |  |
|  | 3 | 1.835 (0.878–3.834) | **2.141 (1.001–4.577)** | **2.415 (1.118–5.217)** | **2.298 (1.015–5.201)** |  |
| **PIV** | 1(Ref) | 1.00 | 1.00 | 1.00 | 1.00 | 0.069 |
|  | 2 | 1.865 (0.918–3.790) | 1.963 (0.961–4.010) | 1.953 (0.954–3.999) | 1.898 (0.885–4.070) |  |
|  | 3 | 1.875 (0.922–3.811) | 1.977 (0.966–4.044) | **2.206 (1.070–4.551)** | 2.070 (0.961–4.460) |  |

Notes:

Model 1: Adjusted for age and gender.

Model 2: Additionally adjusted for clinical thrombosis factors (coronary heart disease, heart failure, hypertension, diabetes, stroke)

Model 3: Further adjusted for paroxysmal atrial fibrillation, oral anticoagulant use, left atrial diameter, and left ventricular ejection fraction

Abbreviations: OR, odds ratio; CI, confidence interval; NLR, neutrophil-to-lymphocyte ratio; MLR, monocyte-to-lymphocyte ratio; PLR, platelet-to-lymphocyte ratio; WMR, white blood cell-to-mean platelet volume ratio; NMR, neutrophil-to-mean platelet volume ratio; SII, systemic immune inflammation index; SIRI, systemic inflammation response index; PIV, pan-immune-inflammation value
